# Geometry-Driven Fabrication of Mini-Tablets via 3D Printing: Correlating Release Kinetics with Polyhedral Shapes

**DOI:** 10.3390/pharmaceutics16060783

**Published:** 2024-06-08

**Authors:** Young-Jin Kim, Yu-Rim Choi, Ji-Hyun Kang, Yun-Sang Park, Dong-Wook Kim, Chun-Woong Park

**Affiliations:** 1 College of Pharmacy, Chungbuk National University, Cheongju 28160, Republic of Korea; 7777ytrewq@gmail.com (Y.-J.K.); choiyurim0730@gmail.com (Y.-R.C.); 2 School of Pharmacy, Institute of New Drug Development, and Respiratory Drug Development Research Institute, Jeonbuk National University, Jeonju 54896, Republic of Korea; jhkanga@jbnu.ac.kr; 3 Research & Development Center, P2K Bio, Cheongju 28160, Republic of Korea; parkys@p2kbio.co.kr; 4 Collge of Pharmacy, Wonkwang University, Iksan 54538, Republic of Korea; pharmengin@gmail.com

**Keywords:** 3D printer, fused deposition modeling (FDM), filaments, hot melt extrusion (HME), mini-tablets, polyhedrons, surface area (SA), release kinetics model

## Abstract

The aim of this study was to fabricate mini-tablets of polyhedrons containing theophylline using a fused deposition modeling (FDM) 3D printer, and to evaluate the correlation between release kinetics models and their geometric shapes. The filaments containing theophylline, hydroxypropyl cellulose (HPC), and EUDRAGIT RS PO (EU) could be obtained with a consistent thickness through pre-drying before hot melt extrusion (HME). Mini-tablets of polyhedrons ranging from tetrahedron to icosahedron were 3D-printed using the same formulation of the filament, ensuring equal volumes. The release kinetics models derived from dissolution tests of the polyhedrons, along with calculations for various physical parameters (edge, SA: surface area, SA/W: surface area/weight, SA/V: surface area/volume), revealed that the correlation between the Higuchi model and the SA/V was the highest (R^2^ = 0.995). It was confirmed that using 3D- printing for the development of personalized or pediatric drug products allows for the adjustment of drug dosage by modifying the size or shape of the drug while maintaining or controlling the same release profile.

## 1. Introduction

Three-dimensional printing of drugs offers revolutionary advantages, such as the possibility of personalizing patient treatment strategies and combining different drugs and release technologies by easily tailoring dosages based on drug shape, size, and release characteristics [1,2,3,4]. Currently, the most pharmaceutically attractive aspects of 3D printing include the prospect of developing low-dose drugs with narrow therapeutic windows and increasing the awareness of pharmacogenomics [5,6]. Additionally, it can improve the dosing flexibility of fixed-dose combination products, which has been a growing requirement for first-line drug therapy for hypertension and infectious diseases (tuberculosis, human immunodeficiency virus) [7,8,9]. The global market for 3D-printed drugs is rapidly growing at a compound annual growth rate (CAGR) of 15.2% from 2021 to 2027. In response, the FDA established guidelines for 3D printing medical device products in 2017. However, there are still technical challenges in 3D printing applications. Also, regulations and guidelines are lacking, especially regarding personalized 3D-printed drugs [3,9].

Fused deposition modeling (FDM)—a type of 3D printing technology—is especially appealing for pharmaceutical solid dosage forms because of its low cost, precise and reproducible control of printed shapes, and feasibility for industrial and laboratory scales. The FDM 3D printer heats the extruded polymeric filament and passes it through a 3D printing nozzle to the build plate. The 3D printing nozzle deposits filaments layer by layer in the XY dimensions, creating a 3D object on the build plate, while simultaneously lowering the build plate to enable bottom-up assembly of the object [10,11,12]. The main method for preparing drug-loaded filaments for use in typical FDM 3D printers is hot melt extrusion (HME) technology. In the HME process, active pharmaceutical ingredients (APIs) are blended with thermoplastic polymers, melted below their glass transition temperature, and subsequently extruded by screws in the HME [13,14]. There are several challenges faced during the preparation of drug-loaded filaments for HME and commercial 3D printing; for example, the original filaments, available for commercial 3D printing, are mainly composed of acrylonitrile butadiene styrene (ABS) and polylactic acid (PLA). However, recent studies are underway to determine and evaluate suitable polymers for oral solid drugs, since ABS and PLA are not appropriate pharmaceutical-grade polymers [15,16]. Another challenge to the preparation of filaments through HME is the filament thickness, which should be 1.75 mm to load commercial FDM 3D printers.

A potential advantage of FDM 3D printers is that they can be used to fabricate tablets with any geometric shape: adjusting the shape and size of the tablet is the simplest way to personalize drug therapy. Many studies have evaluated drug release using various tablet shapes. The evaluation of the dissolution behavior of pellets with cross and clover leaf forms was found to be a complex function of the surface geometry [17]. Donut- and parabolic-shaped tablets were tested to obtain zero-order drug release [18,19], and triangular, cylindrical, and half-spherical tablets were studied for differences in drug release mechanisms [20]. However, the recently developed 3D printing technology was able to easily fabricate and evaluate more sophisticated and diverse shapes than powder compaction. In addition, 3D printing can fabricate shapes that are impossible to produce using powder compaction; a study that evaluated the effect of geometry on the drug release profile for various 3D printing shapes (cube, pyramid, cylinder, and torus), confirmed that surface area/volume (SA/V) is an important control factor for release profiles [21]. 

The most common method of drug administration in children, who are incapable of swallowing solid dosage forms, is to administer crushed tablets or opened gelatin capsules. In such cases, masking and underdosing are impossible, and the desired dissolution profile of the original drug may change because of the increased surface area. Mini-tablets are considered proper oral solid forms intended for children. Mini-tablets manufactured via 3D printing can be easily reduced in size, and the surface area of the tablets can be accurately controlled, as 3D printing allows for dissolution profile adjustments through size conversion [22,23,24,25].

Release kinetics modeling in drug delivery has great potential to facilitate product development and evaluation, especially for patient-personalization or pediatric drug development [26,27,28,29]. To develop a patient-personalized or pediatric drug product using 3D printers, it is necessary to alter the size or shape of an existing product while maintaining the same release profile [30,31,32,33]. In this research, we studied whether 3D printers can elaborately fabricate mini-tablets containing theophylline. Additionally, we evaluated the correlation between release kinetics models and geometric shapes by fabricating polyhedron mini-tablets for the first time to determine whether it is suitable for patient-personalization or pediatric drug development.

## 2. Materials and Methods

### 2.1. Materials

The drug, theophylline, and lubricant, stearic acid, were purchased from Sigma-Aldrich (Gillingham, UK). EUDRAGIT RS PO (EU) was donated by Evonik Industries (Darmstadt, Germany). Hydroxypropyl cellulose (HPC; Klucel HF) was obtained from Ashland (Schaffhausen, Switzerland). PLA filaments (control) were purchased from BeyondTech (BeyondTech, Seoul, Republic of Korea). Anhydrous monobasic potassium phosphate was from Georgiachem (Merck Korea, Seoul, Republic of Korea), and sodium hydroxide (Samchun, Pyeongtaek-si, Republic of Korea) and tetrahydrofuran (Daejung Chemicals, Siheung-si, Republic of Korea) were used for dissolution tests and high-performance liquid chromatography (HPLC) analysis. Acetonitrile (HPLC grade) was purchased from Honeywell Burdick and Jackson (Muskegon, MI, USA). All the experiments were performed using Milli-Q (Millipore, Billerica, MA, USA) distilled water.

### 2.2. Preparation of Theophylline-Loaded Filaments via HME

The compositions of the drug, polymer, and plasticizer mixtures are detailed in Table 1. Anhydrous theophylline and stearic acid were passed through a 355 μm sieve to remove aggregates. All ingredients were accurately weighed to ensure a batch size of 100 g, and powder blends were mixed using a tubular mixer^®^ (T2F; Willy A. Bachofen Maschinenfabrik, Muttenz, Switzerland) at 49 rpm for 30 min. Prior to HME, half of the mixture was dried using a laboratory oven for 30 min at 50 °C. All mixtures (pre-dried and non-pre-dried) were separately loaded and extruded using a twin-screw extruder (Process11; Thermo Fisher Scientific, Karlsruhe, Germany) at 170 °C and screw speed of 50 rpm through a 2.00-mm thick circular die. The polymer melts were guided into a winder (Filawinder; Filastruder, Snellville, GA, USA) to fine-tune the filament thickness, and the thickness was measured by distinguishing between the long and short axes using a digital caliper (CD-20APX, Mitutoyo Corp., Kawasaki, Japan).

### 2.3. Moisture Uptake

The moisture uptake of each pre-dried or non-pre-dried mixture was examined by measuring the mass loss on drying (LOD) of the samples using a moisture-analyzing balance (AND MF-50 Moisture Analyzer; A&D Instruments, Abingdon, UK) in triplicate.

### 2.4. Three-Point Bend Test

The extruded filaments were tested for their mechanical properties using a three-point bend test with a micro-tester (Instron 5848; Instron Corporation, Norwood, MA, USA). Filament samples and a commercial PLA filament (control for extruded filament comparison) were cut to a length of 50 mm. A digital caliper was used to measure the thickness of the samples, which were then placed in the center of two lower support beams with a gap of 15 mm.

The measurements were initiated when the trigger force exceeded 100 N and reached a speed of 1 mm/s. Measurements were repeated five times for each formulation, and Bluehill software (version 2.0; Instron Corporation, Norwood, MA, USA) was used for data analysis.

### 2.5. Three-Dimensional Printing for Mini-Tablet of Polyhedron Shapes

Mini-tablets were fabricated using a fused-deposition modeling 3D printer (Pro 2 Dual 3D printer; Raise 3D, Irvine, CA, USA) with drug-loaded filaments with the same batch of TP2 formulation. Prior to the printing step, 3D models were designed using Rhinoceros CAD software (version 4.0, Robert McNeel, Seattle, WA, USA) and exported as a stereolithography file into IdeaMaker software (version 3.4.2, Raise 3D, Irvine, CA, USA).

The printer settings were as follows: high resolution with a printer nozzle thickness of 0.2 mm and the raft option activated, using the other nozzle with PLA; an extrusion temperature of 210 °C, infill speed during extrusion of 30 mm/s, shell width of 0.8 mm, and a layer height of 0.05 mm; an infill percentage to produce high-density tablets of 96% and a triangular infill pattern type. The sizes of the mini-tablets were controlled using the scale function of the software to fabricate a consistent mini-tablet volume.

### 2.6. Physicochemical Characterizations

#### 2.6.1. Differential Scanning Calorimetry (DSC)

The thermal response of pure substances (theophylline, HPC, EU, and stearic acid), physical mixtures (PM), filaments, and mini-tablets were analyzed using a DSC thermal analyzer system (DSC Q2000; TA Instruments, New Castle, DE, USA). For DSC measurement, the filament and mini-tablet samples fabricated with the TP2 formulation were prepared by crushing them with a mortar and pestle. The PM was also prepared with the composition of TP2 formulation. All samples were accurately weighed to 4 mg and loaded into aluminum pans, then analyzed at temperatures ranging from 0 to 300 °C at a heating rate of 10 °C/min with nitrogen purge gas (flow rate: 50 mL/min). The thermal response of the prepared sample was calculated using TA universal analysis advantage software (version 5.2.6., TA Instruments, New Castle, DE, USA).

#### 2.6.2. Powder X-ray Diffraction (PXRD)

PXRD was used to assess the crystalline state of theophylline in the PM, filaments, and mini-tablets. The filament and mini-tablet samples fabricated with the TP2 formulation were prepared by crushing them with a mortar and pestle. The PXRD patterns of the samples were analyzed using a D8 Discover with GADDS (Bruker AXS, Billerica, MA, USA) with an ASC glass sample holder (18 × 0.5). Then, 2θ scans were conducted between 5° and 60° with a wavelength of 1.54 Å and a Cu radiation source of (40 kV, 40 mA) at room temperature.

#### 2.6.3. Fourier Transform Infrared Spectroscopy (FT-IR)

Infrared spectra were obtained using an FT-IR spectrometer (IFS 66v/S; Bruker Optics, Ettlingen, Germany) to investigate the possible interactions between the drug and selected excipients in their PM, crushed filaments, and mini-tablets. The spectrum was collected at wavelengths of 4000–650 cm^−1^ using an attenuated total reflection (ATR) accessory with a ZnSe crystal with 32 scans, a resolution of 4.00 cm^−1^, and a speed of 5 kHz.

### 2.7. Scanning Electron Microscopy (SEM)

The side and cross-sectional morphologies of the drug-loaded filaments (pre-dried and non-pre-dried) were assessed using SEM (Ultra Plus; Carl Zeiss, Jena, Germany) operating at 3.00 kV and SE2 signal. In addition, mini-tablets of various shapes were examined by SEM; all the samples were attached to SEM stubs using double adhesive tape and then coated with a white-gold film (600 Å) using sputter deposition prior to imaging.

### 2.8. Characterization of Tablets Morphology

A digital caliper was used to measure the length of one edge of each mini-tablet and a Quantum FX XRCT instrument (Perkin Elmer, Waltham, MA, USA) was used to double-check the measured edge using a digital caliper. It was equipped with a microfocus X-ray tube, L10101 (Hamamatsu Photonics, Hamamatsu, Japan), and flat panel detector (PaxScan 1313; Varian Medical Systems, Palo Alto, CA, USA). Finally, the mini-tablets were weighed, and their surface areas (SA) and volumes (V) were calculated based on these morphological dimensions.

### 2.9. Drug Content Analysis and Dissolution Tests

The filaments were ground with a mortar and pestle and then accurately weighed at 100 mg. The filament samples were dissolved 8 h after sonification for 1 h in pH 6.0 phosphate buffer. The dissolved samples were filtered through a 0.45 μm PVDF syringe filter (Whatman, GE healthcare Co. Ltd., Chicago, IL, USA) and content analysis of the dissolved samples was performed using HPLC (U3000, Thermo Fisher Scientific, Waltham, MA, USA) equipped with a Capcell Pak C8 column (4.6 mm × 250 mm, 5 μm). The mobile phase was loaded at a flow rate of 1.2 mL/min and comprised of water-THF–acetonitrile: 0.1% THF in water, pH 8.0 adjusted with 0.1 M NaOH; and acetonitrile (90:10, *v*/*v*). The injection volume was 50 μL and the stop time was 8 min per sample. The wavelength was set at 273 nm in a column oven at 25 °C. The method showed linearity between 0.5 and 32 μg/mL, with R^2^ = 0.9999 and limits of detection and quantitation of 0.126 and 0.381 μg/mL, respectively. The accuracy was 100.4 ± 0.01% and 99.7 ± 4.43% at 8.0 and 1.0 μg/mL concentrations, respectively.

The in vitro release rate of theophylline from filaments or mini-tablets was evaluated in triplicate using a USP dissolution test apparatus II (VK-7010; Varian Medical Systems, Palo Alto, CA, USA). The filament samples were prepared by cutting it into approximately 100 mg to match the weight of the mini-tablets being produced. The dissolution evaluation was conducted at a paddle speed of 50 rpm for 21 h in 900 mL of pH 6.8 phosphate buffer medium. This dissolution method was a simplified and modified version of the Dissolution Test 1 from the Theophylline Extended-Release Capsules of USP to suit the purposes of this study. The samples were collected from each dissolution vessel at 5 mL, filtered through a 0.45 μm PVDF syringe filter, and subsequently analyzed using an HPLC-UV method under the same chromatographic conditions as the drug content analysis. To investigate the dissolution behavior, the mini-tablets were transferred to a Petri dish at predetermined intervals and photographed using a camera (DSC-RX100M6; Sony, Tokyo, Japan).

### 2.10. Release Kinetics Studies

To analyze the in vitro release data, various models were used to describe the release kinetics [34]:

- Zero-order model

The zero-order model describes a system in which the drug release rate is independent of its concentration:

(1) $$Q_{t}=K_{0}t,$$

where *Q* is the amount of drug released or dissolved, *Q*_0_ is the initial amount of drug in the solution, *K*_0_ is the zero-order rate constant, and *t* is the time.

- First-order model

This model describes the absorption and removal of some drugs, which depends on the concentration of the drug:

(2) $$\log C=\log C_{0}-\frac{K_{1}t}{2.303}\;,$$

where *C*_0_ is the initial concentration of the drug, *K*_1_ is the first-order constant, and *t* is the time.

- Hixon-Crowell model

This model describes the release of the dose from a system based on the cubic root of the surface area and diameter of the particle or tablet [35]:

(3) Q013−Qt13=KHC·t,

where *Q_t_* is the amount of drug released at time *t*, *Q*_0_ is the initial amount of drug in the tablet, and *K_HC_* is the rate constant for the Hixson–Crowell rate equation.

- Higuchi model

Higuchi described a mathematical equation for the release of drugs from an insoluble matrix as the square root of a time-dependent process based on the Fickian diffusion equation [36]:

(4) Q=KH·t12

where *K_H_* is the rate constant for the Higuchi rate equation.

## 3. Results and Discussion

### 3.1. Preparation of 3D-Printable Filaments

**Figure 1:**
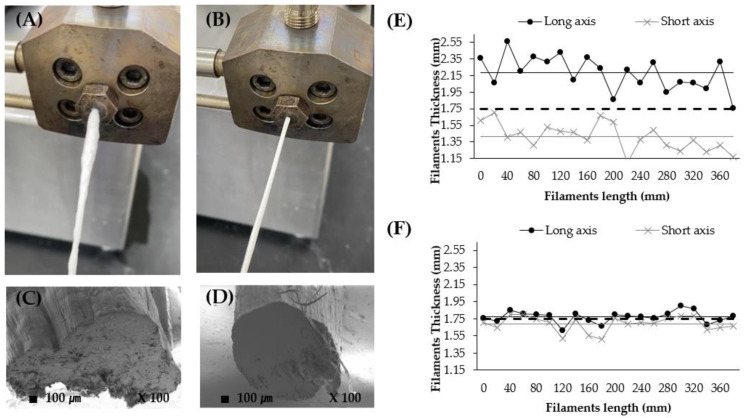
Photographs of filament being extruded from the HME (**A**,**B**), SEM images of the cross-section of extruded filament (**C**,**D**), and sequential filament thickness of the long and short axes, measured at intervals of 20 mm. ((**A**,**C**,**E**) non-pre-drying, (**B**,**D**,**F**) pre-drying).

Formulations with different HPC/EU ratios containing 20% *w*/*w* theophylline and 2.5% stearic acid (Table 1) were extruded from the HME and adjusted in thickness using a winder. All formulations, according to the polymer ratio, were extruded without exceeding the upper limit of the die pressure or extruder torque under HME conditions (170 °C and screw speed of 500 rpm). The thickness of HME-extruded filaments affects the process during which commercial filaments are loaded into FDM 3D printers, and therefore plays a critical role in printability [37]. In other words, regardless of the polymer ratio, the thickness of the filament containing theophylline should be 1.75 ± 1.00 mm. To prevent moisture evaporation during filament extrusion, which may cause a deviation in thickness, PLA was dried before extrusion. The LOD values of theophylline, HPC, EU, and their PM before pre-drying were 0.95 ± 0.13%, 2.55 ± 0.22%, 2.05 ± 0.15%, and 2.93 ± 0.38%, respectively (*n* = 3). After pre-drying for 30 min, LOD values of theophylline, HPC, EU, and PM were 0.82 ± 0.14%, 1.58 ± 0.13%, 1.45 ± 0.09%, and 1.32 ± 0.10%, respectively (*n* = 3). There was no difference in theophylline LOD values before and after pre-drying; it was therefore assumed that the moisture in PM was due to the polymers, HPC and EU. Additionally, the LOD values of the pre-dried PM for 60 min and 120 min were similar at 1.29 ± 0.06%, and 1.28 ± 0.12%, respectively, indicating that free moisture is rapidly dried within approximately 30 min. Consequently, the pre-drying of the powder mixtures for 30 min before extrusion effectively inhibited the generation of bubbles, referred to as die swell, which formed to be caused by the evaporation of free moisture in the filaments (Figure 1A). This further resulted in rough and curved surfaces of the extruded filament (Figure 1C). Conversely, the extrusion process after pre-drying yielded no bubbles in the extruded filament, which appeared to have drained the moisture out in advance, owing to pre-drying. Additionally, the surface of the pre-dried filaments was smooth, and the cross-section was closer to the circle, not as ellipse as the non-pre-dried filaments (Figure 1B,D) [38,39].

Filament thickness was measured every 20 mm in length (Figure 1E,F). The target thickness of a commercially available PLA filament was 1.75 mm, as indicated by the dotted lines in Figure 1. Filament thickness deviated more from the target value in non-pre-dried filaments than in pre-dried filaments (Figure 1E). The large deviation was caused by the bubbles escaping in one direction, which created the long and short axis in the non-pre-dried filaments. The average thicknesses of the long and short axes were 2.18 ± 0.21 mm and 1.41 ± 0.16 mm, respectively. Additionally, the ratio of the long and short axes in non-pre-dried filaments was 1.56 ± 0.21 (Table 2). In 3D printing, a filament with a large thickness is squeezed or broken in the gear section and cannot be pushed down to the nozzle. A thin filament will bend in a spiral while descending if it is not given a consistent force from the gear in the direction of the nozzle [40]. Therefore, non-pre-dried filaments cannot be used in 3D printing. Moreover, Figure 1B shows that the thickness of the pre-dried filament nearly corresponds to the target thickness of 1.75 mm: the average thicknesses of the long and short axes were 1.77 ± 0.07 mm and 1.69 ± 0.09 mm, respectively, and the ratio of the long and short axes was 1.05 ± 0.03. Through pre-drying, it was shown that the RSD % and error % to 1.75 mm of the thickness along the filament length was smaller and closer to the target thickness (Table 2). This means an improvement in the uniformity of printing, without issues such as an inability to insert filament into the gear section or filament breakage (Table 2). 

### 3.2. Texture Analysis

Five pre-dried filaments with different HPC/EU ratios containing 20% (*w*/*w*) of theophylline with appropriate thicknesses were prepared to ensure that they could be properly loaded into the 3D printer. For successful printing, the filament should be flexible, while simultaneously being appropriately rigid and soft [16,41]. Brittle filaments (too rigid) are broken by the force of the 3D printer feeding gear, and too-soft filaments become wedged between the driving gears [40,42]. The 3-point bending flexural test was used to assess the flexure stress, extension, and load of the material. These measurements help predict the 3D printability of filaments. The stiffness of the extruded filaments was calculated from the flexure load and extension values obtained from a 3-point bending test [16,37].

Table 3 shows the results of 3-point bending flexural tests for the extruded TP filaments and PLA filaments. The PLA filament had the highest flexure stress and extension, so it could predict the rigidness and softness of the material. The PLA was well-bent and appropriately rigid, and could therefore print without becoming wedged between the gears. In contrast, TP5, which has only EU as a polymer, had the lowest flexure stress and extension, which meant that it could also be easily broken with low strain. Because of its brittleness, the TP5 was nearly unable to print, as it was broken by the 3D printer gears.

The mechanical properties between TP1 and TP2, in which HPC was the dominant filament property, were not significantly different. The flexure extension values of TP1 and TP2 were 3.92 ± 0.92 mm and 4.26 ± 0.58 mm, respectively, while the flexure stress values were 39.22 ± 6.50 MPa and 40.33 ± 3.86 MPa, respectively. As a result, they were fairly soft, but compared to the issues observed for PLA in the gears, the softness of TP1 and TP2 would not pose problems. The extension of TP3 and TP4 decreased as the EU decreased in the HPC/EU ratio, and the stress increased compared to TP1 and TP2. The flexure extension values for TP3 and TP4 were 2.86 ± 0.17 mm and 2.77 ± 0.53 mm, respectively, while the flexure stress values were 80.89 ± 8.93 MPa and 90.77 ± 8.61 MPa, respectively. Therefore, their softness and rigidity had the best properties for 3D printing among the TP formulations. 

### 3.3. Filaments Dissolution

**Figure 2:**
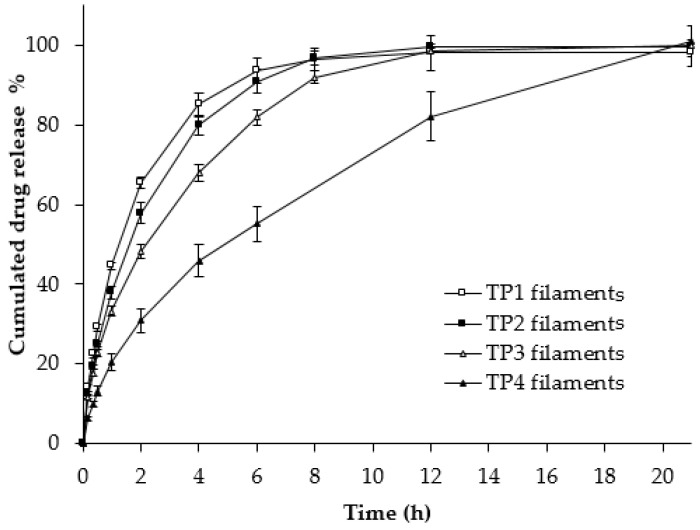
Drug release profiles of the TP filaments in phosphate buffer (pH 6.0). Each of the values represents the mean ± standard deviation (*n* = 3).

Filament dissolution studies were performed on 3D-printable filaments that were pre-dried and extruded. Filaments with different release profiles were successfully prepared, as shown in Figure 2. A highly water-soluble API can disperse in two polymer matrices to form solid dispersions during the HME process, while sustaining the release of API in a phosphate buffer medium at pH 6.0. Higher HPC in the HPC/EU ratio led to faster drug release rates. Drug release of formulations TP1 and TP2 were 85.18 ± 2.93% and 79.77 ± 2.36% at 4 h, respectively. While TP3 released 81.90 ± 1.89% at 6 h, TP4 released 82.14 ± 6.22% at 12 h. Both HPC and EU follow pH-independent swelling mechanisms. However, HPC is soluble, while EU is insoluble; consequently, higher EU in TP3 and TP4 resulted in more sustainable drug release and swelling time, allowing it to retain its original shape for longer. In this study, the mini-tablets were 3D-printed with a TP2 filament, which had an HPC/EU ratio of 6:1. TP2 was expected to swell at a reasonable time, making it easier to check the dissolution patterns before and after swelling. 

### 3.4. Physicochemical Characterizations

**Figure 3:**
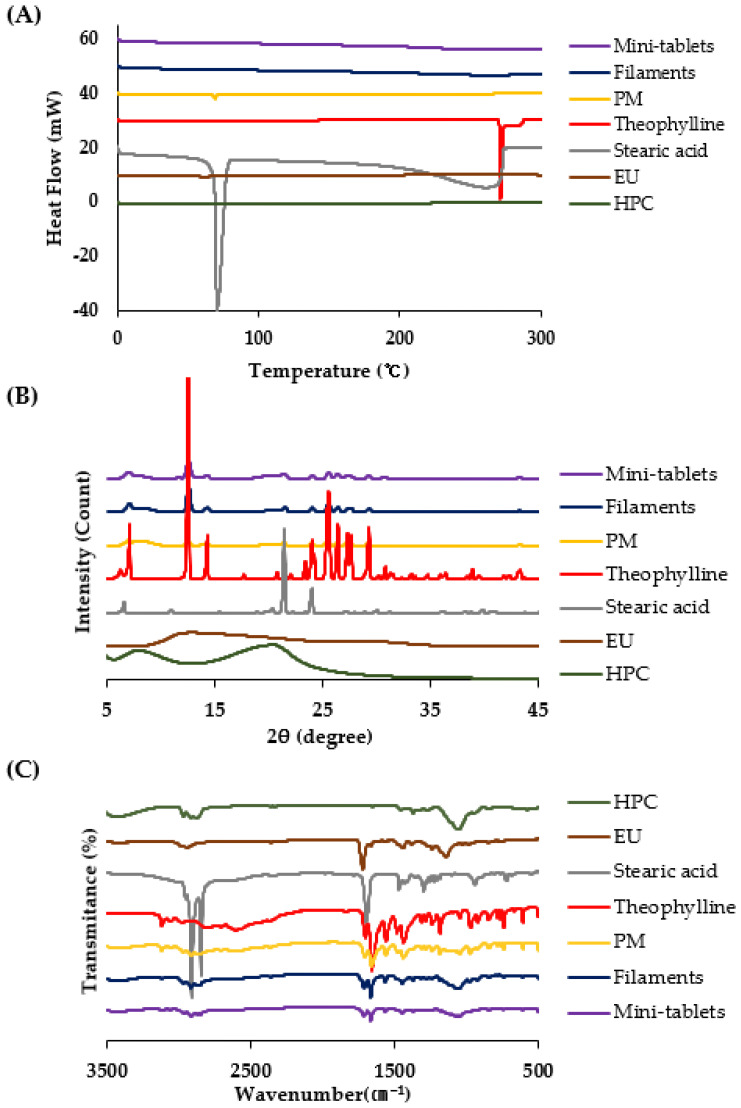
(**A**) DSC thermal curves, (**B**) PXRD curves, and (**C**) FT-IR spectra of HPC, EU, stearic acid, theophylline, PM, filaments, and mini-tablets.

DSC was conducted to examine the changes in the crystallinity of the bulk materials, extruded filament, and 3D printing tablet during the thermal processes. As shown in Figure 3A and Figure S1), theophylline exhibited a strong endothermic peak at approximately 271 °C, corresponding to its melting point [43,44]. The decomposition temperature of pure theophylline was found to be around 285 °C following its melting point [45]. This suggests that theophylline decomposition did not occur at the processing temperatures of 170 °C and 210 °C for the filaments and mini-tablets, respectively. However, with the introduction of HPC and EU, the endothermic peak of theophylline was not clearly observed in the DSC curves of the PM or the filaments and subsequent mini-tablets. As previously reported [46,47], the absence of these peaks in the DSC curves indicates that theophylline might be solid dispersed/solubilized in the polymer matrix during the DSC experiment for the PM or during the HME and 3D printing processes.

To further confirm the thermal properties of the samples, a PXRD analysis was conducted. The high temperatures employed during HME and 3D printing can degrade thermolabile drugs and polymers. As illustrated in Figure 3B and Figure S2, theophylline showed numerous sharp diffraction peaks, with unique peaks at 2θ = 7.1°, 12.4°, 14.3°, and 24.0°, which corresponded to the known diffraction patterns of monohydrate crystal form [48]. Surprisingly, the PXRD results for PM, filaments, and mini-tablets were considerably different from the results obtained from the DSC curves. PM, filaments, and mini-tablets showed numerous peaks with reduced intensities, suggesting that theophylline crystals were converted to a partially crystalline state through molecular dispersion into the polymer matrix. One possible explanation for this observation is the effects of homogeneously mixing the active material and amorphous polymer under the thermal processing conditions of HME and 3D printing. However, in this study, no significant thermal degradation was observed for either the filaments or the mini-tablets.

FT-IR evaluation of the main drug peaks was used to assess chemical reactions during the HME and 3D printing processes. The findings showed that the peaks of all functional groups remained intact without shifting, disappearing, or moving. The characteristic peaks of the pure drug were compared with the peaks obtained after FT-IR evaluation of PM, filaments, and mini-tablets. Similar characteristic peaks appeared with minor differences at 3054 cm^−1^, 2981 cm^−1^ (N-H broad bend), 1658 cm^−1^ (N-H bending), 1560 cm^−1^ (N-H stretching), and 1702 cm^−1^ (C=O stretching) for theophylline and for the others (Figure 3C and Figure S3) [49,50]. Therefore, the drug is in a free state and there is no interaction between the drug and the polymers used.

### 3.5. Three-Dimensional printing and Morphological Characterization of Mini-Tablets

**Figure 4:**
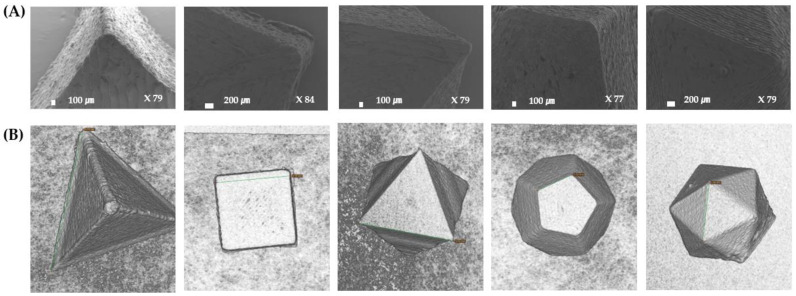
Morphology images of the 3D-printed polyhedron mini-tablets (from left: tetrahedron, hexahedron, octahedron, dodecahedron, icosahedron). (**A**) SEM images and (**B**) Micro-CT images.

It was possible to print mini-tablets with various polyhedrons using a 3D printer at 210 °C on the printer nozzle loaded with a theophylline-supplemented filament (Figure 4). Five polyhedron mini-tablets were printed by modeling the same volume: 97.34 mm^3^. Therefore, in order to have the same volume, the length of one side of each of the polyhedrons was modeled as follows: tetrahedron, 9.38 mm; hexahedron, 4.60 mm; octahedron, 5.91 mm; dodecahedron, 2.33 mm; icosahedron, 3.55 mm. 

The length of one side of the printed polyhedron mini-tablet was measured with a caliper (*n* = 3) and the measured lengths of the tetrahedron, hexahedron, octahedron, dodecahedron, and icosahedron were 9.05 ± 0.14 mm, 4.60 ± 0.06 mm, 5.91 ± 0.0 5 mm, 2.35 ± 0.03 mm, and 3.58 ± 0.02 mm, respectively. In addition, when the same samples were examined using X-ray micro-tomography (micro-CT) and the length of one edge in the software was measured, the length was similar to that measured with the caliper (Figure 4B). Among the five polyhedrons, the tetrahedrons showed the greatest deviation in the side length between the samples because of the limitation of the FDM method. The limitations of the 3D printer’s 0.2 mm nozzle caused a deviation in the tetrahedrons because the last layer to be laminated corresponds to the vertex of the tetrahedron.

The polyhedrons that were modeled to corresponding volumes slightly differed in weight. Weight deviation within a range of 5% was also observed in the corresponding polyhedron mini-tablets. Despite pre-drying to maintain consistent filament thickness, the slight variation in thickness still affects the extrusion amount during 3D printing, leading to weight deviation that impacts reproducibility. Nevertheless, the edges or angles of the polyhedrons were otherwise exquisitely printed, as observed in the micro-CT or SEM images (Figure 4A,B). In the case of the tetrahedron, the angle of the vertex was not exactly 60° and was slightly round because it had to be one layer, while the others were constructed by overlapping layers. In addition, depending on the way the layers accumulated, mini-tablets were assumed to have different weights. The tetrahedron and hexahedron weighed less than the others because the size of the upper layer did not exceed that of the lower layer. Therefore, when printed, it appears that the lower layer was cut off by the printer nozzle. The volume, density, SA, V, SA/V, and SA/W were calculated based on the measured weight and length of one edge of the polyhedrons (Table 4). Mathematical calculations indicate that for polyhedrons with the same volume, the SA increases as the number of edges increases (the number of faces increases), that is, as they neared the sphere. The SA values calculated from the edge of the fabricated polyhedrons also followed the same trend as the mathematical calculations, showing an increase in SA with an increasing number of faces.

### 3.6. In Vitro Release Studies

The drug content of the TP2 filaments used in the dissolution test was evaluated using HPLC, and the result was 96.6 ± 0.8%, indicating low variation. This suggested that the drug was well-dispersed in the polymer and no drug was degraded during the process. The drug release rate from water-soluble and swellable polymers is governed by the relative contributions of two mechanisms: drug diffusion and polymer dissolution (surface erosion) [51]. In addition, the drug release profiles of various 3D-printed geometries were studied, and it was suggested that the SA to V ratio is directly related [21]. The purpose of this dissolution study was to eliminate the effects of volume-induced drug release and identify only the effects of SA. By using swelling polymers (HPC and EU), the erosion of the tablet was minimized, reducing the effect of other factors that affect drug release, excluding SA.

**Figure 5:**
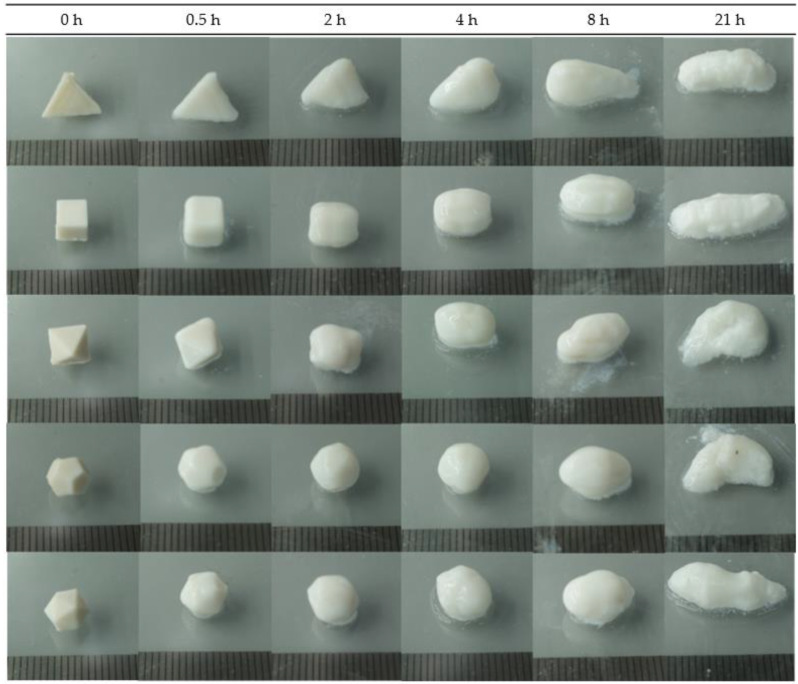
Photographs of 3D-printed mini-tablets by dissolution times. (From top: tetrahedron, hexahedron, octahedron, dodecahedron, and icosahedron).

**Figure 6:**
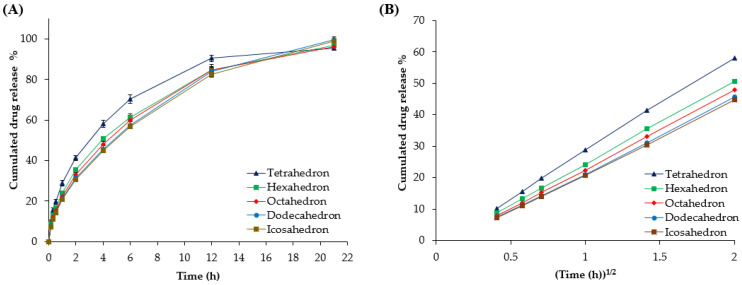
(**A**) Drug release profiles and (**B**) Higuchi (square root) kinetics profiles of the polyhedrons in phosphate buffer (pH 6.0).

As shown in Figure 5, all mini-tablets (tetrahedron, hexahedron, octahedron, dodecahedron, and icosahedron) retained their respective shapes for up to 4 h, and then swelled into similar forms after 6 h, after which it was difficult to distinguish them by shape. As the polyhedron mini-tablets swelled over time, the differences in drug release rates among the polyhedrons decreased, with the drug release at 6 h being 58.01 ± 1.72%, 50.68 ± 0.95, 47.88 ± 1.55%, 45.57 ± 1.69%, and 44.73 ± 0.11%, respectively. Furthermore, at 12 h, when the polyhedrons were fully swollen and indistinguishable in each shape, the drug release rates among the polyhedrons became even more similar (Figure 5 and Figure 6A). Therefore, the drug release patterns of all mini-tablets until 6 h were fitted with various release kinetics models and were well-fitted by the Higuchi model (R^2^ = 0.9992 ± 0.0003, 0.9997 ± 0.0003, 0.9998 ± 0.0001, 0.9996 ± 0.0003, and 0.9997 ± 0.0002, respectively; Table 5 and Figure 6B); this confirmed that drug release was time-dependent, based on Pician’s diffusion equation on SA [52,53]. Additionally, the Hixon–Crowell model, which describes the dissolution rate based on the surface area and diameter of particles or tablets, did not exhibit the highest correlation. This outcome is likely due to the swelling (not erosion) and sustained release characteristics of the polymer excipients used in this study, resulting in the Higuchi model being more suitable. 

**Figure 7:**
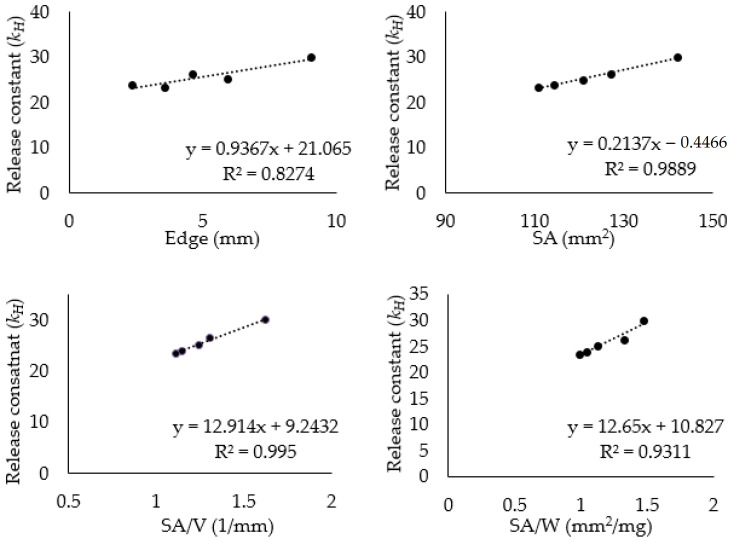
Various physical parameters (edge, SA, SA/V, SA/W) of polyhedrons fitted to the release constant (*K_H_*) of the Higuchi model.

Furthermore, the characteristics of various polyhedron shapes (edge, SA, SA/V, and SA/W) were correlated with the release kinetic constant (*K_H_*) of the Higuchi model (Figure 7). First, the R^2^ in SA for *K_H_* was 0.9889, suggesting that *K_H_* increased at a constant rate depending on the SA of the mini-tablets. Although the polyhedrons with the same volume and weight were modeled, the volume and weight of the polyhedron were slightly different, so the R^2^ values for SA/V and SA/W were 0.9950 and 0.9311, respectively. The ideally designed polyhedron mini-tablets should have the same volume and density since they were manufactured with the same filament. However, the resolution of the 3D printer caused variations in weight rather than volume, leading to a decreased correlation of SA/W compared to SA/V. If 3D-printed mini-tablets were elaborately fabricated in a set size, there would be no difference between the R^2^ of the SA and the above two values.

In this study, the SA of the polyhedron was obtained using the length of one side of the fabricated polyhedron. In the case of the tetrahedron, because the last layer of the FDM 3D printer was a point, not one surface, the length of one side could not be printed as a set parameter; thus, the SA was calculated as the length of one side, which was bound to deviate from the actual SA [54]. It can also be confirmed that the correlation of the R^2^ value with its *K_H_* is better in SA than SA/V [55].

Interestingly, the R^2^ value for the length of one edge of the mini-tablets was 0.8274, with respect to *K_H_*. In polyhedrons of the same volume, the greater the number of edges, the shorter the edge length (i.e., the closer the sphere or the smaller the SA): the length of one edge is formed in accordance with the circumscribed sphere; the tetrahedron contains one edge in half of the circumference; the hexahedron and octahedron contain two; and the dodecahedron and icosahedron have three. However, the edge is replaced by a diagonal or the height of each surface, depending on the characteristics of the polyhedrons. For these reasons, the edge slightly correlates with *K_H_*, though not as strongly as with SA. 

This study confirmed that the drug release profile can be controlled or predicted by modifying the dimensions of the tablet’s SA [56]. Drawing a calibration curve for release kinetics and SA means that, in addition to the tablet being designed to have a patient-personalized dissolution profile, mini-tablets for children can be designed with the same profile without sectioning or crushing the tablets during administration [40,57].

## 4. Conclusions

Theophylline-loaded filaments were successfully developed to the target thickness through a pre-drying process to avoid the formation of water bubbles prior to extrusion. The filaments with various HPC/EC ratios showed suitable mechanical and printing properties for the 3D printing of mini-tablets, except for TP5, which contained only EU. Polyhedron mini-tablets (tetrahedron, hexahedron, octahedron, dodecahedron, and icosahedron) were fabricated using a 3D printer to corresponding volumes to avoid affecting volume-induced drug release profiles. In the dissolution study, the mini-tablets’ shapes were maintained for up to 6 h in phosphate buffer (pH 6.0), after which they lost their shape due to swelling of the polymer matrices. The drug release patterns of all mini-tablets after 6 h were well-fitted with the Higuchi model. Consequently, the *K_H_* of each mini-tablet was fitted with the edge, SA, SA/V, and SA/W, in which R^2^ was calculated. SA has an important effect on drug release as long as it retains its shape; therefore, SA modification can control drug release while maintaining the original weight of the tablet. In addition, calculating the SA and kinetics of a 3D-printed tablet can predict its drug release profile [58,59]. However, despite the Higuchi model showing the highest correlation in this study, it is important to recognize that different dissolution kinetic models may be selected for drugs of different BCS classes or for formulations with different release mechanisms. Therefore, further research appears necessary to advance the development of personalized 3D-printed drugs.

## Figures and Tables

**Table 1:** The compositions of the mixtures (% *w*/*w*). HPC: Hydroxypropyl cellulose, EU: EUDRAGIT RS PO.

	Theophylline (% *w*/*w*)	HPC (% *w*/*w*)	EU (% *w*/*w*)	Stearic Acid (% *w*/*w*)
TP1	20.0	77.5	-	2.5
TP2	20.0	66.3	11.3	2.5
TP3	20.0	51.7	25.9	2.5
TP4	20.0	38.8	38.8	2.5
TP5	20.0	-	77.5	2.5

**Table 2:** Filament (non-pre-drying and pre-drying) thickness properties of the long and short axes (*n* = 20).

Filaments Thickness		Average of Thickness (mm)	RSD to Average (%)	Average of Error to 1.75 mm (%)	Long/Short Ratio
Non-pre-drying	Long axis	2.18 ± 0.21	9.45%	24.63 ± 11.77%	1.56 ± 0.21
	Short axis	1.41 ± 0.16	11.57%	19.40 ± 9.33%	
Pre-drying	Long axis	1.77 ± 0.07	3.93%	3.31 ± 2.41%	1.05 ± 0.03
	Short axis	1.69 ± 0.09	5.20%	4.31 ± 4.14%	

**Table 3:** Results of 3-point bending tests for the extruded filaments (TP), with PLA as reference.

(*n* = 5)	Maximum Flexure Load (N)	Maximum Flexure Extension (mm)	Maximum Flexure Stress (MPa)	Maximum Flexure Strain (%)	Modulus (MPa)	Property
TP1	7.25 ± 1.66	3.92 ± 0.92	39.22 ± 6.50	19.88 ± 3.63	1215.00 ± 163.68	Adequate
TP2	5.33 ± 0.33	4.26 ± 0.58	40.33 ± 3.86	19.50 ± 2.74	1428.20 ± 196.05	Adequate
TP3	11.72 ± 0.73	2.86 ± 0.17	80.89 ± 8.93	13.52 ± 0.99	2508.67 ± 406.25	Adequate
TP4	14.46 ± 0.87	2.77 ± 0.53	90.77 ± 8.61	13.58 ± 3.10	2422.80 ± 228.93	Adequate
TP5	5.24 ± 0.41	1.12 ± 0.05	28.28 ± 2.88	5.72 ± 0.36	2329.40 ± 169.20	Brittle
PLA	20.81 ± 0.26	6.61 ± 1.29	149.87 ± 3.08	30.74 ± 6.05	3694.20 ± 121.47	Adequate

**Table 4:** Physical properties of 3D-printed polyhedron mini-tablets calculated with measured edge and weight.

	Weight (mg)	Volume (mm^3^)	Density (mg/mm^3^)	Edge (mm)	SA (mm^2^)	SA/V (1/mm)	SA/W (mm^2^/mg)
Tetrahedron	96.87 ± 4.77	87.49 ± 4.09	1.107 ± 0.013	9.05 ± 0.14	141.99 ± 4.44	1.624 ± 0.026	1.467 ± 0.030
Hexahedron	96.27 ± 2.91	97.59 ± 4.06	0.987 ± 0.019	4.60 ± 0.06	127.16 ± 3.54	1.304 ± 0.018	1.321 ± 0.017
Octahedron	107.83 ± 5.86	97.16 ± 2.49	1.109 ± 0.039	5.91 ± 0.05	120.86 ± 2.06	1.244 ± 0.011	1.123 ± 0.047
Dodecahedron	110.37 ± 6.58	99.91 ± 3.87	1.104 ± 0.027	2.35 ± 0.03	114.35 ± 2.96	1.145 ± 0.015	1.038 ± 0.038
Icosahedron	112.30 ± 1.32	99.83 ± 1.28	1.125 ± 0.002	3.58 ± 0.02	110.79 ± 0.95	1.110 ± 0.005	0.987 ± 0.004

**Table 5:** Statistical parameters of various polyhedrons obtained after fitting the drug release data to various release kinetics models.

Formulation	Zero-Order		First-Order		Hixson–Crowell		Higuchi	
	R^2^	*k*_0_	R^2^	*k*_1_	R^2^	*k_HC_*	R^2^	*k_H_*
Tetrahedron	0.9172 ± 0.0051	13.23 ± 0.33	0.9894 ± 0.0011	0.04 ± 0.00	0.9811 ± 0.0017	0.26 ± 0.01	0.9992 ± 0.0003	30.14 ± 0.73
Hexahedron	0.9278 ± 0.0070	11.59 ± 0.24	0.9898 ± 0.0031	0.03 ± 0.00	0.9835 ± 0.0036	0.21 ± 0.01	0.9997 ± 0.0003	26.40 ± 0.62
Octahedron	0.9345 ± 0.0033	10.99 ± 0.37	0.9905 ± 0.0011	0.03 ± 0.00	0.9848 ± 0.0013	0.20 ± 0.01	0.9998 ± 0.0001	25.14 ± 0.87
Dodecahedron	0.9386 ± 0.0045	10.49 ± 0.37	0.9911 ± 0.0018	0.03 ± 0.00	0.9859 ± 0.0022	0.19 ± 0.01	0.9996 ± 0.0003	24.00 ± 0.85
Icosahedron	0.9377 ± 0.0015	10.28 ± 0.06	0.9904 ± 0.0015	0.02 ± 0.00	0.9852 ± 0.0019	0.18 ± 0.00	0.9997 ± 0.0002	23.53 ± 0.19

## Data Availability

Data sharing is contained within the article and Supplementary Materials.

## Supplementary Materials

The following supporting information can be downloaded at: https://www.mdpi.com/article/10.3390/pharmaceutics16060783/s1, Figure S1. DSC thermal curves of Theophylline, PM, Filaments, and Mini-tablets. Figure S2. PRXD curves of Theophylline, PM, Filaments, and Mini-tablets. Figure S3. FT-IR spectra of Theophylline, PM, Filaments, and Mini-tablets.
