# Supplementary material for: Geometry-Driven Fabrication of Mini-Tablets via 3D Printing: Correlating Release Kinetics with Polyhedral Shapes
Source: Pharmaceutics. 2024 Jun 8;16(6):783. doi: 10.3390/pharmaceutics16060783 (PMC11207496; doi:10.3390/pharmaceutics16060783)
Supplement: Supplementary file 1 [file pharmaceutics-16-00783-s001.zip › pharmaceutics-3039049-supplementary.pdf]

pharmaceutics

Article

Geometry-driven fabrication of mini-tablets via 3D-printing: correlating release kinetics with polyhedral shapes

Young-Jin Kim [1], Yu-Rim Choi [1], Ji-Hyun Kang [2], Yun-Sang Park [3], Dong-Wook Kim [4] and Chun-Woong Park [1,*]

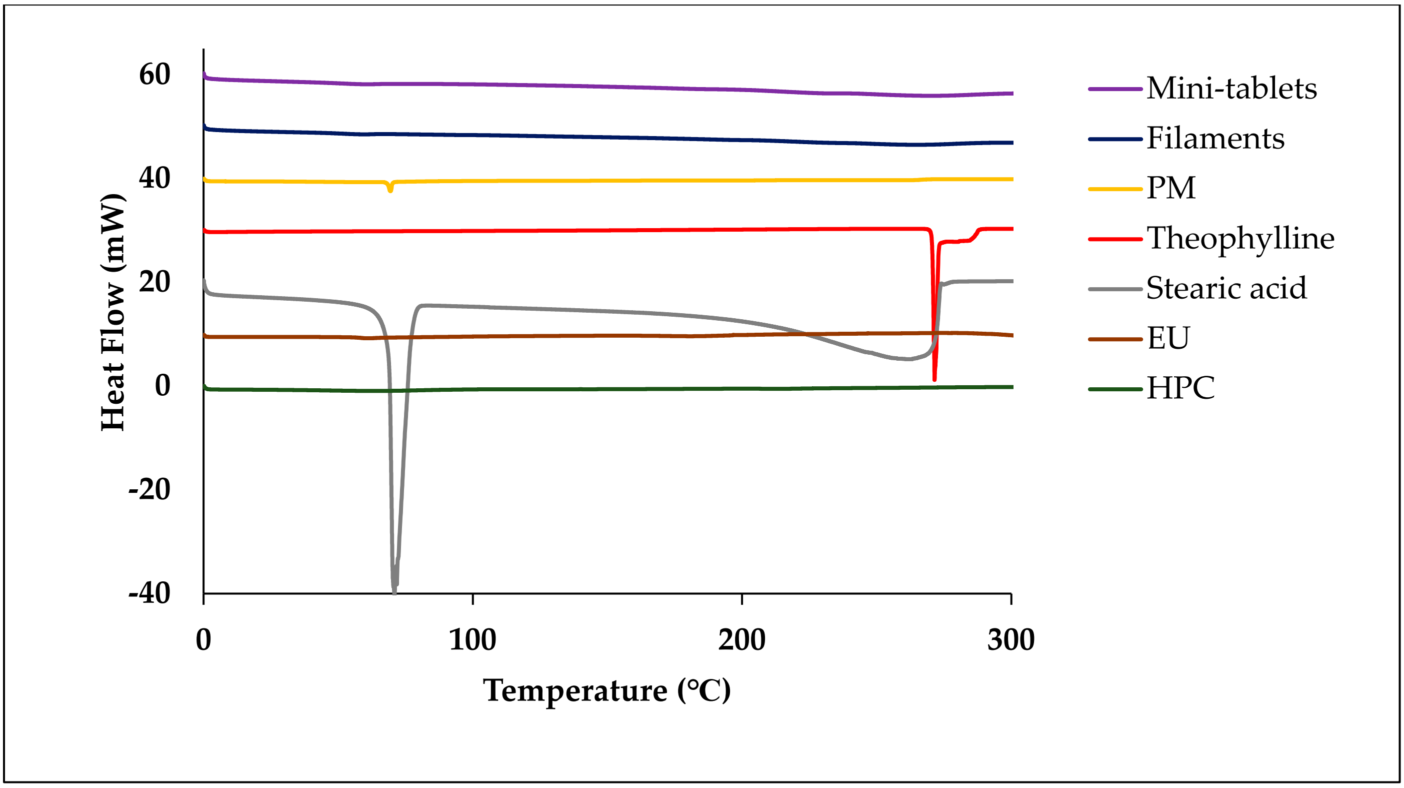

Figure S1. DSC thermal curves of Theophylline, PM, Filaments, and Mini-tablets

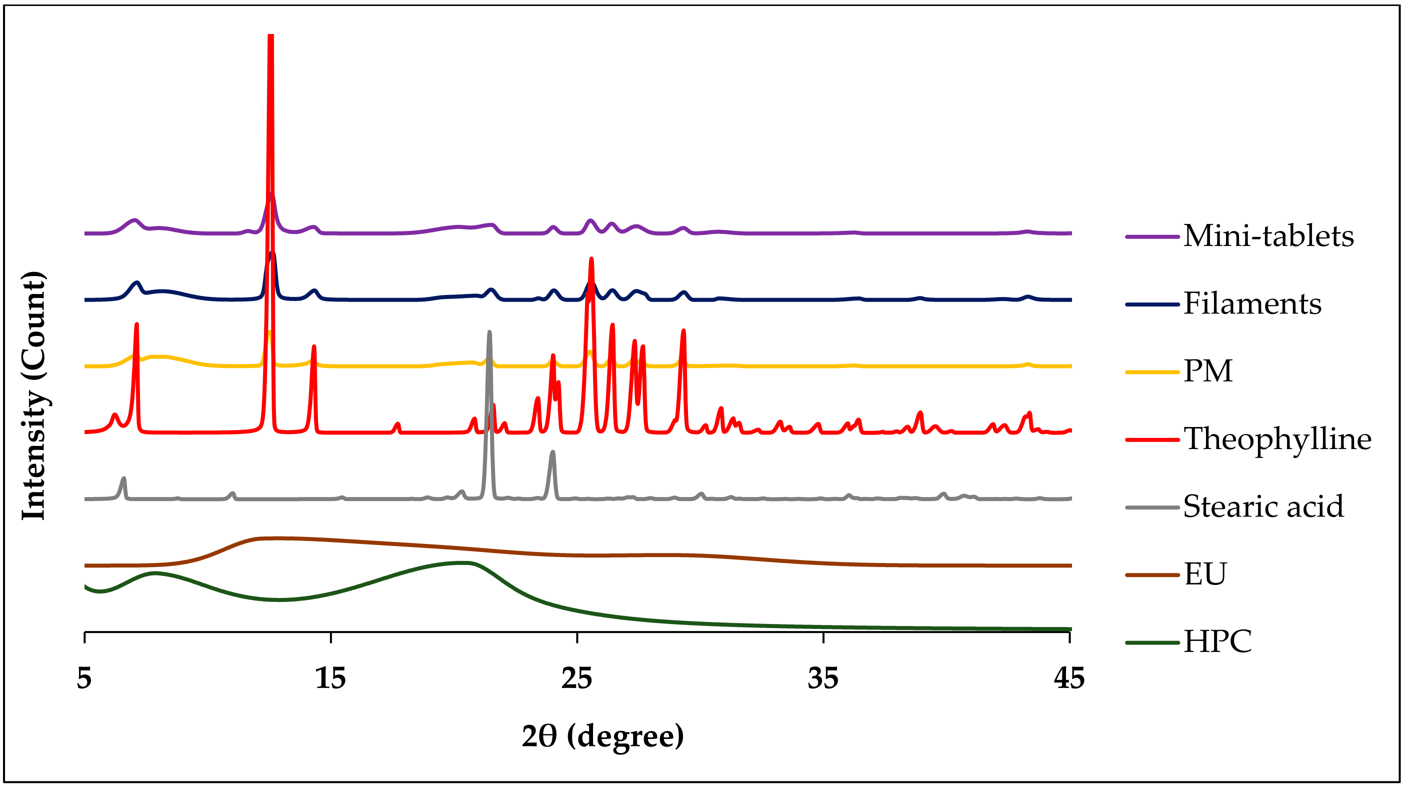

Figure S2. PRXD curves of Theophylline, PM, Filaments, and Mini-tablets

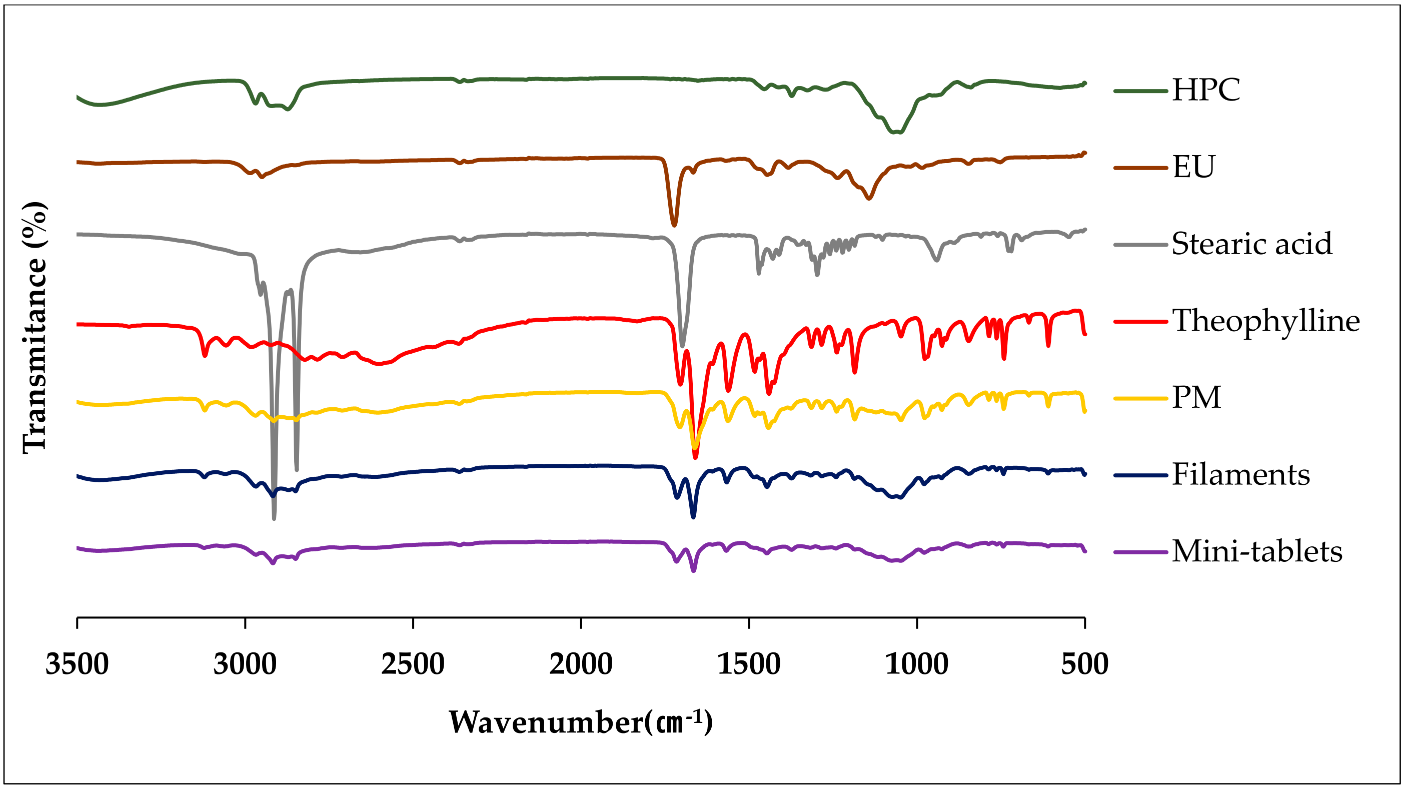

Figure S3. FT-IR spectra of Theophylline, PM, Filaments, and Mini-tablets
